# Efficacy and safety of the biosimilar denosumab candidate (Arylia) compared to the reference product (Prolia®) in postmenopausal osteoporosis: a phase III, randomized, two-armed, double-blind, parallel, active-controlled, and noninferiority clinical trial

**DOI:** 10.1186/s13075-022-02840-8

**Published:** 2022-06-30

**Authors:** Ahmadreza Jamshidi, Mahdi Vojdanian, Mohsen Soroush, Mahmoud Akbarian, Mehrdad Aghaei, Asghar Hajiabbasi, Zahra Mirfeizi, Alireza Khabbazi, Gholamhosein Alishiri, Anousheh Haghighi, Ahmad Salimzadeh, Hadi Karimzadeh, Fatemeh Shirani, Mohammad Reza Hatef Fard, MohammadAli Nazarinia, Soosan Soroosh, Nassim Anjidani, Farhad Gharibdoost

**Affiliations:** 1 Rheumatology Research Center, Shariati Hospital, Tehran University of Medical Sciences, Tehran, Iran; 2 Rheumatology Research Center, Tehran University of Medical Sciences, Tehran, Iran; 3 Rheumatology Department, AJA University of Medical Sciences, Tehran, Iran; 4 Golestan Rheumatology Research Center (GRRC), Golestan University of Medical Sciences, Gorgan, Iran; 5 Department of Rheumatology, Guilan Rheumatology Research Center, School of Medicine, Razi Hospital, Guilan University of Medical Sciences, Rasht, Iran; 6 Rheumatic Diseases Research Center, Mashhad University of Medical Sciences, Mashhad, Iran; 7 Connective Tissue Diseases Research Center, Tabriz University of Medical Sciences, Tabriz, Iran; 8 Chemical Injuries Research Center, Systems Biology and Poisonings Institute, Baqiyatallah University of Medical Sciences, Tehran, Iran; 9 Rheumatology Department, Iran University of Medical Sciences, Tehran, Iran; 10 Rheumatology Research Center, Sina Hospital, Tehran University of Medical Sciences, Tehran, Iran; 11 Department of Internal Medicine, School of Medicine, Isfahan University of Medical Sciences, Isfahan, Iran; 12 Department of Rheumatology, Iran University of Medical Sciences, Tehran, Iran; 13 Rheumatology Ward, Internal Medicine Department, Mashhad University of Medical Sciences, Mashhad, Iran; 14 Shiraz Geriatric Research Center, Shiraz University of Medical Sciences, Shiraz, Iran; 15 Rheumatology Department, AJA University of Medical Sciences, Tehran, Iran; 16 Medical Department, Orchid Pharmed Company, Tehran, Iran

**Keywords:** Denosumab, Arylia, Prolia®, Osteoporosis, Biosimilar

## Abstract

**Background/objective:**

Osteoporosis is a global health concern with an increasing prevalence worldwide. Denosumab is an antiresoptive agent that has been demonstrated to be effective and safe in osteoporotic patients. This study aimed to compare the efficacy and safety of the biosimilar denosumab candidate (Arylia) to the originator product (Prolia®) in postmenopausal osteoporotic patients.

**Methods:**

In this randomized, double-blind, active-controlled, noninferiority trial, postmenopausal osteoporotic patients received 60 mg of subcutaneous Arylia or Prolia® at months 0, 6, and 12 and were followed up for 18 months. The primary endpoint was the noninferiority of the biosimilar product to the reference product in the percentage change of bone mineral density (BMD) in 18 months at the lumbar spine (L_1_-L_4_), total hip, and femoral neck. The secondary endpoints were safety assessment, the incidence of new vertebral fractures, and the trend of bone turnover markers (BTMs).

**Results:**

A total of 190 patients were randomized to receive either biosimilar (*n* = 95) or reference (*n* = 95) denosumab. In the per-protocol (PP) analysis, the lower limits of the 95% two-sided confidence intervals of the difference between Arylia and Prolia® in increasing BMD were greater than the predetermined noninferiority margin of − 1.78 at the lumbar spine, total hip, and femoral neck sites (mean differences [95% CIs] of 0.39 [− 1.34 to 2.11], 0.04 [− 1.61 to 1.69], and 0.41 [− 1.58 to 2.40], respectively). The two products were also comparable in terms of safety, new vertebral fractures, and trend of BTMs.

**Conclusion:**

The efficacy of the biosimilar denosumab was shown to be noninferior to that of the reference denosumab, with a comparable safety profile at 18 months.

**Trial registration:**

ClinicalTrials.gov, NCT03293108; Registration date: 2017–09-19.

**Supplementary Information:**

The online version contains supplementary material available at 10.1186/s13075-022-02840-8.

## Introduction

Osteoporosis is a common metabolic bone disease [1], affecting an estimated more than 200 million people worldwide [2], and is highly prevalent in the elderly population in Iran [3]. It is characterized by diminished bone mineral density (BMD) and deterioration of bone quality (microarchitectural changes), leading to compromised bone strength and an augmented risk of fractures [4, 5]. According to the International Osteoporosis Foundation (IOF), it is estimated that worldwide, approximately 30% of women and 20% of men over the age of 50 develop osteoporosis-induced fractures in their lifetime [6].

Postmenopausal osteoporosis guidelines highly recommend pharmacologic treatment in patients with osteoporosis and patients with osteopenia at high risk for fractures [7, 8]. The initial pharmacologic treatment for most osteoporotic patients at high fracture risk includes denosumab, zoledronate, and teriparatide [7, 9]. Denosumab is a fully human monoclonal antibody that blocks the interaction between receptor activator of nuclear factor kappa-B ligand (RANKL) and its receptor (RANK) on osteoclasts, leading to inhibition of osteoclast formation as well as osteoclast-mediated bone resorption [10, 11]. Denosumab was the first drug to be approved by the US Food and Drug Administration (FDA) with this mechanism and has been used for osteoporosis since 2010 [12]. In the main study of denosumab (FREEDOM study), the drug effectively reduced the risk of new vertebral and nonvertebral fractures and improved the BMD at the lumbar spine and total hip [13].

Considering the global aging population and the expected increase in osteoporosis incidence, developing drugs and preventive approaches are of great importance in managing osteoporosis and its consequences. Biosimilar products are comparable in safety, efficacy, and quality to licensed biological reference products. They are often provided at a lower cost and provide better accessibility in lower-income countries. The preclinical studies of the biosimilar denosumab (Arylia, AryoGen Pharmed, Iran) showed no meaningful difference from the reference product (Prolia®, Amgen Inc., USA). In the present study, we assessed the noninferiority of Arylia to Prolia® and compared their efficacy and safety profiles in postmenopausal osteoporotic patients within 18 months.

## Method

### Study design and participants

This was a double-blind, randomized, active-controlled, two-armed, parallel-group, noninferiority, phase 3 study performed from April 2017 to August 2020 in 12 centers in Iran. Postmenopausal women aged between 45 and 75 years were included in the study if they had a *T* score of ≤  − 2.5 and ≥  − 4 at the lumbar spine (L_1_–L_4_), total hip, or femoral neck or were at high risk for fracture based on the Fracture Risk Assessment Tool (FRAX) criteria [14] and needed medical treatment. Key exclusion criteria included conditions affecting the safety and efficacy of drugs such as malignancy, osteonecrosis of the jaw (ONJ) risk factors (e.g., diagnosis of cancer, poor oral hygiene, periodontal and/or dental diseases, having dentures, and comorbid disorders such as anemia with a hemoglobin level less than 11 g/dl, history of diseases with coagulopathy, oral and dental infections), long-lasting untreated hypocalcemia (albumin-adjusted serum calcium level less than 8 mg/dl), history of recent bisphosphonate treatment (parenteral bisphosphonates in the last 12 months, oral bisphosphonates in the last 3 months), corticosteroid treatment (> 5 mg prednisone daily or equivalent for ≥ 3 months), confined to bed (for two weeks during the past three months), and the impossibility of measuring BMD for any reason.

Other exclusion criteria were as follows: hypersensitivity to denosumab or any component of the formulation; malabsorption syndrome; history of thyroid surgery, parathyroid surgery or intestinal resection if causing malabsorption; chronic kidney disease (CKD) stage 4 or 5 (glomerular filtration rate (GFR) < 30 cc/min); 25-hydroxy vitamin D level less than 20 ng/ml (such patients could be enrolled after management of vitamin D deficiency with two tests showing blood levels above 20 ng/ml within a month); untreated hypercalciuria (> 250 mg/24 h) and hypocalciuria (< 100 mg/24 h); severe and active infections; inability to take 1000 mg elemental calcium as a supplement; conditions affecting bone turnover (e.g., hypo- or hyperparathyroidism, hypo- or hyperthyroidism, hypocalcemia, inflammatory rheumatologic diseases such as rheumatoid arthritis, Paget’s disease of bone, unresponsive osteomalacia, which means not responding to 1-month administration of vitamin D); one severe (> 50% vertebral height loss) or more than two moderate (25–50% vertebral height loss) vertebral fractures; history of severe bone pain with bisphosphonates; use of parathyroid hormone or its derivatives, systemic hormone-replacement therapy, selective estrogen receptor modulator, calcitonin, or calcitriol within six weeks before study enrollment; use of heparin (more than 20,000 international units/day for ≥ 6 months prior to the study); and patients with chronic conditions such as allergies, asthma, and coagulation disorders who required to use corticosteroids (> 5 mg prednisone daily or equivalent for ≥ 3 months) or heparin (more than 20,000 international units/day for 6 months and longer) during the study period.

Written informed consent was obtained from all study patients. The study was approved by the ethics committees of Tehran University of Medical Sciences and Tabriz University of Medical Sciences. The study was registered at Clinicaltrial.gov (NCT03293108).

### Randomization and blinding

Randomization and treatment allocation occurred after primary screenings and confirmation of patients' eligibility. Randomization was carried out centrally using R-CRAN software version 3.2.3 in a 1:1 ratio, with permuted blocks with lengths of two or four. The physicians, patients, and outcome assessors were masked to treatment assignments to prevent bias.

### Procedures

Patients were randomly assigned to receive 60 mg of either biosimilar or reference denosumab subcutaneously every 6 months, including at baseline, month 6, and month 12. Prior to denosumab administration, vitamin D and calcium levels were corrected in patients with deficiency. All patients were supplemented with daily calcium (1000 mg of elemental calcium) and vitamin D (at least 400 IU) during the study. According to the exclusion criteria, all allowed concomitant medications were continued. Concomitant drugs were recorded at baseline and during periodic visits.

Participants underwent periodic assessments at months 0, 1, 3, 6, 9, 12, 15, and 18 of the study. The BMD of the lumbar spine (L_1_–L_4_), total hip, and femoral neck were measured by dual-energy X-ray absorptiometry (DXA) scan (Hologic 4500 or higher) at the screening and last (month 18) visits with the same device. Necessary training for BMD measurement was given to staff before the start of the trial, based on the same guideline approved by the principal investigator and careful monitoring during the trial. To ensure precision, a standard quality control program that involved training, certification, and recertification of DXA operators was implemented in all BMD measurement centers periodically. In addition, DXA devices were assessed and calibrated before and periodically during the study. The same physician and radiologist assessed lateral spine X-ray radiography (T_4_–L_4_) at the screening visit and month 18. The evaluation of bone turnover markers (BTMs), including bone-specific alkaline phosphatase (BSAP), osteocalcin (OC), procollagen type 1 N-terminal pro-peptide (P1NP), serum C-terminal telopeptide (CTX), and serum N-terminal telopeptide (NTX), was performed on fasting blood samples at baseline and during periodic visits. The immunogenicity assessment was performed by ELISA at months 0, 6, 12, and 18.

### Safety assessment

During this study, adverse events (AEs) were monitored at each scheduled visit. Any clinically significant change in physical examination, vital signs, and laboratory data of clinical interest was considered an AE. All AEs were classified based on the Medical Dictionary for Regulatory Activities (MedDRA) terms. The MedDRA terms were also used for addressing the AEs throughout this paper. All the reported events were graded according to the National Cancer Institute Common Terminology Criteria for Adverse Events (CTCAE) v5.0. The causality relation was assessed based on the World Health Organization (WHO) criteria.

Infections and infestations, eczema, ONJ, atypical femoral fracture (AFF), bone fracture, cardiovascular disorder, neoplasm benign, malignant and unspecified (including cysts and polyps), and pancreatitis acute were considered adverse events of special interest (AESIs).

### Outcomes

The primary endpoint was the noninferiority of the biosimilar denosumab to the reference denosumab in improving the percentage change in BMD at the lumbar spine (L_1_–L_4_), total hip, and femoral neck over 18 months of the study. The secondary endpoints included the incidence of new vertebral fractures, adverse events, immunogenicity, and changes in biochemical markers of bone metabolism during the study.

### Statistical analysis

The sample size was calculated using a one-sided independent sample t-test with a 2.5% significance level. A sample size of 95 patients per intervention arm was required to achieve a power of 80% to establish noninferiority for the lumbar spine (L_1_–L_4_) BMD change from baseline at month 18 by considering a drop-out rate of 10% during the trial. In one study, the efficacy of Prolia® in comparison with placebo for lumbar spine BMD improvement was reported to be 7.1% [15]. The margin of noninferiority was set at − 1.78 based on calculation and clinical considerations. The populations were assumed to have equal standard deviations of 4.116. The biosimilar denosumab would be noninferior to the reference product if the lower limit (L_L_) of the 95% confidence interval (95% CI) of the between-group difference in the percent BMD change after 18 months, calculated by a two-sample *t*-test, was greater than the predetermined noninferiority margin of − 1.78.

To conduct sensitivity analysis for the primary endpoint (percent change in BMD), an ANCOVA model was performed considering baseline BMD values and treatment groups as covariates. The least-square means and 95% CIs were calculated based on the ANCOVA model. All primary analyses were performed using both per-protocol (PP) and intention-to-treat (ITT) sets. The missing BMD values at the 18-month timepoint were imputed based on a linear regression model including BMD and serum NTX baseline values, and patients with missing values for baseline serum NTX were not imputed in ITT analysis. The PP set was defined as all patients with no major protocol violations. The ITT set was defined as all randomized patients who received at least one dose of the study drug.

The incidence of new vertebral fractures was analyzed by the chi-square test, and the trends of the BTMs were compared with the longitudinal analysis using the GEE model (with an exchangeable working correlation matrix) adjusting corresponding values at baseline as covariates. The safety set included all randomized patients who received at least one dose of the study drug. Safety evaluation was reported as the incidence rate of AEs, and between-group differences in incidence rates were assessed by the chi-squared test. All statistical analyses were conducted using STATA version 14.0 and R Version 3.2.3 or later.

## Results

**Fig. 1:**
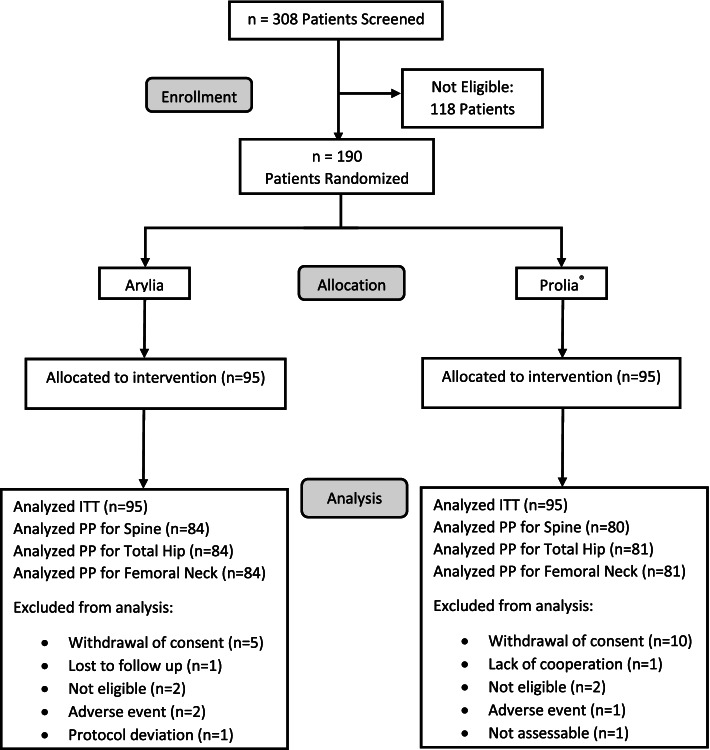
Patient flow diagram

A total of 308 patients were screened, of which 118 were excluded, and 190 patients fulfilled the study eligibility criteria. Ninety-five patients were randomly assigned to each study group, receiving biosimilar or reference denosumab (Fig. 1). The baseline characteristics and demographics of the patients are presented in Table 1.

**Table 1:** Baseline characteristics of participants

**Variable**	**Arylia **(*N* = 95)	**Prolia**® (*N* = 95)
Age, year	61.59 (5.94)	60.60 (6.62)
Height, cm	153.14 (5.88)	154.59 (5.74)
Weight, kg	66.44 (11.28)	64.72 (9.88)
Body-mass index, kg/m^2^	28.35 (4.69)	27.10 (4.09)
*T* score, SD		
Spine (L_1_–L_4_)	− 3.09 (0.54)	− 3.10 (0.54)
Total hip	− 1.27 (0.83)	− 1.29 (0.87)
Femoral neck	− 1.84 (0.83)	− 1.88 (0.68)
25 (OH) vitamin D3, ng/mL	41.80 (19.48)	46.53 (22.15)
Albumin-adjusted serum calcium, mg/dL	9.15 (0.55)	9.10 (0.53)
Previous bisphosphonate use	6	2

Data are mean (SD)

### BMD changes

BMD changes were evaluated in 164 patients (84 patients in the Arylia group and 80 in the Prolia® group) at the lumbar spine (L_1_–L_4_) and in 165 patients (84 patients in the Arylia group and 81 in the Prolia® group) at the total hip and femoral neck. At month 18, there were 24 patients with missing BMD at the total hip and femoral neck and 25 patients with missing BMD at the lumbar spine. The corresponding reason for missing BMD values is provided in Fig. 1. One patient in the Arylia group was excluded from the PP population because of protocol deviation, and the BMD was not missing at month 18. The mean (SD) baseline BMD at the lumbar spine, total hip, and femoral neck were 0.71 (0.06), 0.79 (0.10), and 0.64 (0.09), respectively, in the Arylia group and 0.71 (0.06), 0.78 (0.11), and 0.64 (0.08), respectively, in the Prolia® group. The mean (SD) final (month 18) BMD at the lumbar spine, total hip, and femoral neck were 0.75 (0.07), 0.80 (0.09), and 0.66 (0.08), respectively, in the Arylia group and 0.75 (0.07), 0.81 (0.10), and 0.65 (0.08), respectively, in the Prolia® group. No significant difference was noticed in baseline and final BMD values at either site between the two study groups (*p* = 0.85, 0.90, and 0.75 at the baseline visit and *p* = 0.92, 0.83, and 0.64 at the final visit at the lumbar spine, total hip, and femoral neck, respectively).

**Fig. 2:**
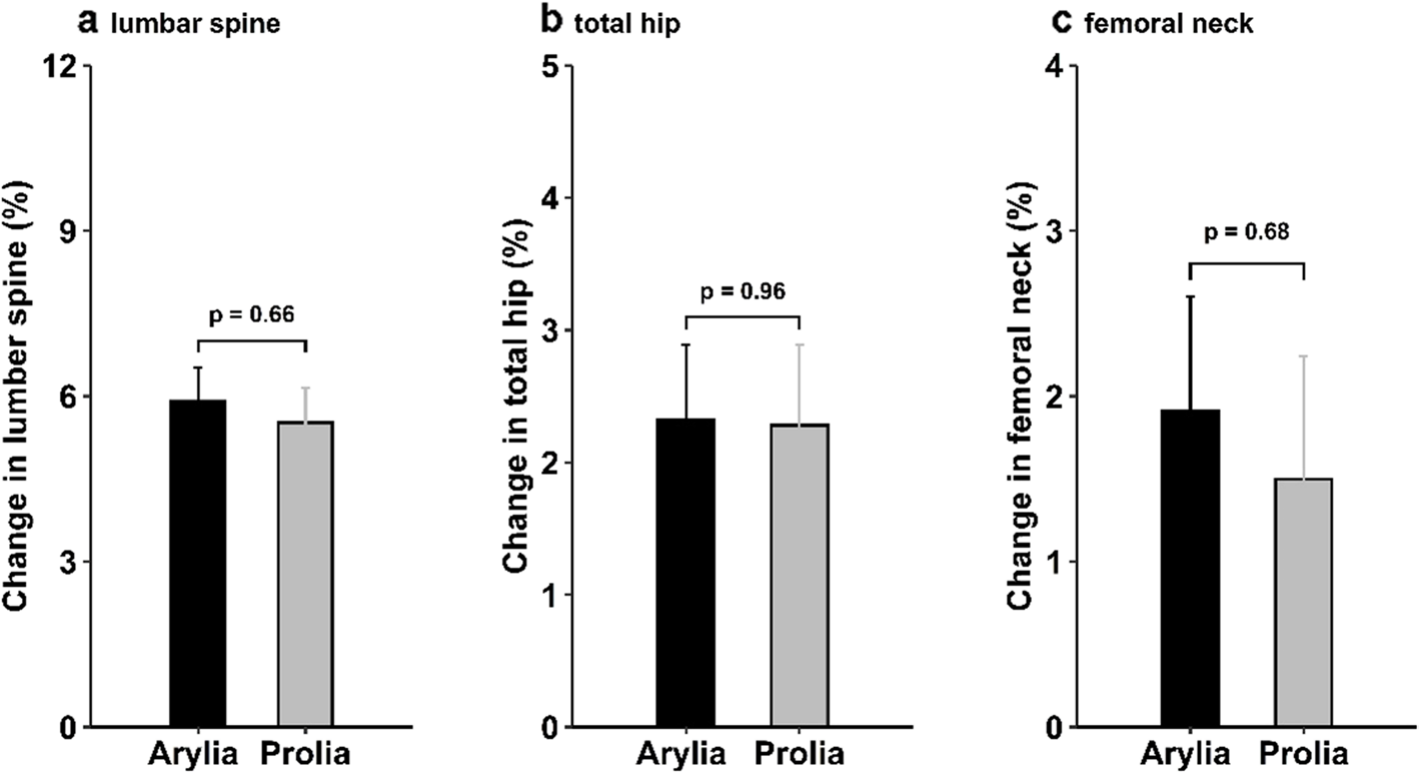
Mean percentage change in BMD of the lumbar spine (L_1_–L_4_) (**a**), total hip (**b**), and femoral neck (**c**). Error bars show standard error. The plot is based on the PP set

The mean (SD) percent changes in BMD at the lumbar spine, total hip, and femoral neck were 5.91 (5.58), 2.32 (5.24), and 1.91 (6.32), respectively, in the Arylia group and 5.52 (5.59), 2.28 (5.52), and 1.50 (6.62), respectively, in the Prolia® group in the PP set. The differences between the treatment groups were not statistically significant at either site (*p* = 0.66, 0.96, and 0.68, respectively; Fig. 2). Detailed comparisons of the mean percent changes in BMD at all measured sites in the PP and ITT analysis sets are provided in Additional file 1.

In the ANCOVA, the least-squared mean (SE) percent changes in BMD at the lumbar spine, total hip, and femoral neck were 5.89 (0.60), 2.28 (0.54), and 1.94 (0.67), respectively, in the Arylia group and 5.53 (0.62), 2.31 (0.55), and 1.46 (0.68), respectively, in the Prolia® group in the PP set. The differences between the treatment groups were not statistically significant at either site (*p* = 0.67, 0.97, and 0.62, respectively). Detailed comparisons of least-squared mean percent changes in BMD at all measured sites in PP and ITT analysis sets are provided in Additional file 2.

### Noninferiority

**Fig. 3:**
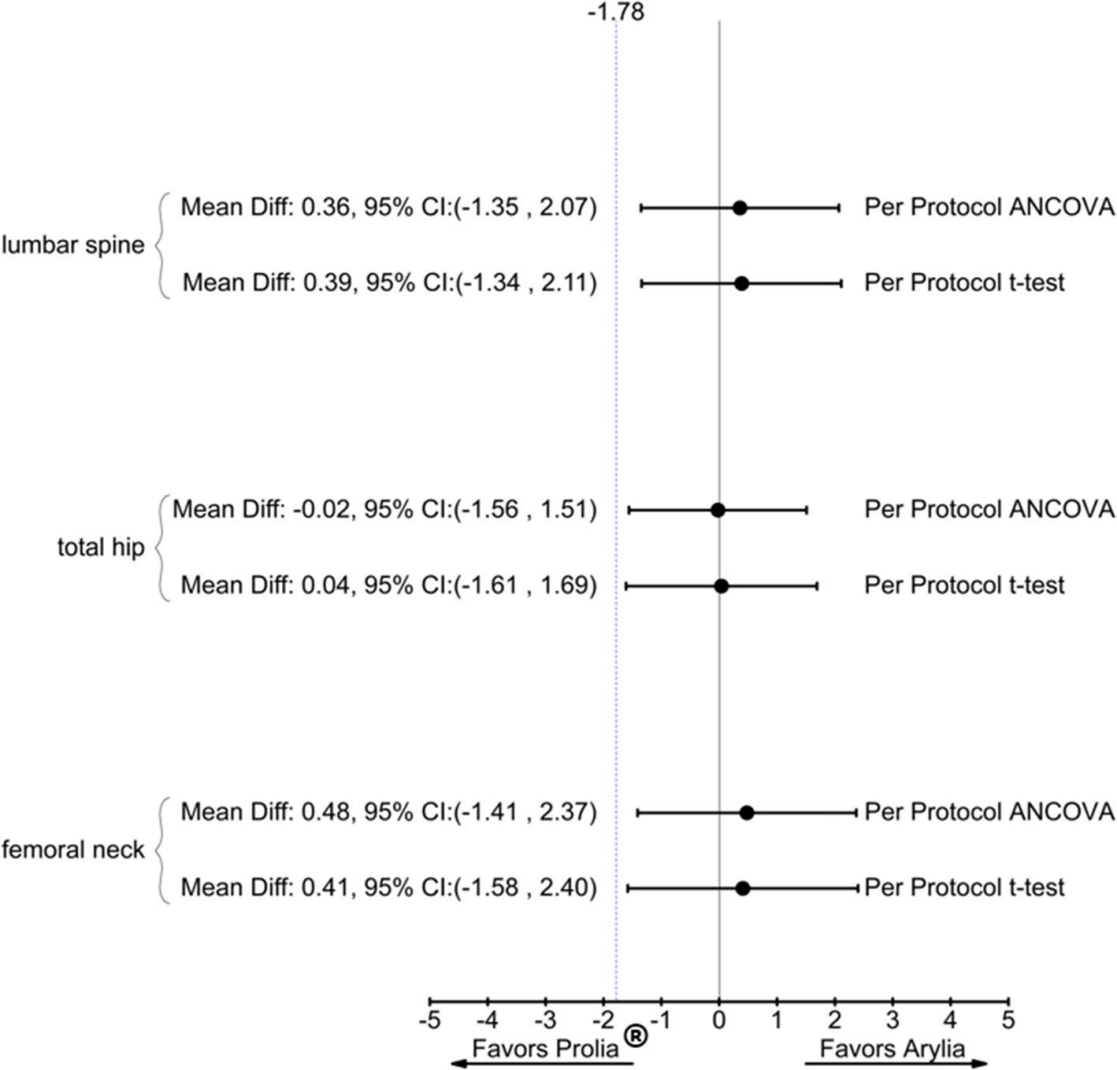
Forest plot for comparing Arylia versus Prolia® in terms of mean percent changes in BMD of the lumbar spine (L_1_–L_4_), total hip, and femoral neck at 18 months of the study. Forest plot demonstrating both *t* test analysis and ANCOVA model for PP set

The primary endpoint of the study was met, as the lower limits of 95% two-sided confidence intervals of differences in the mean percent changes in BMD at the lumbar spine, total hip, and femoral neck were all greater than the predefined margin of − 1.78 in the PP analysis set (Fig. 3). The results were similar when we adjusted the data for baseline BMD in the ANCOVA (Fig. 3). Similarly, the lower limits of 95% CIs of differences in the mean percent changes in BMD at the lumbar spine and femoral neck were greater than the − 1.78 margin in the ITT analysis set. The results for the ITT analysis set are provided in Additional file 3.

### New vertebral fractures

Regarding the occurrence of new vertebral fractures, no new vertebral fractures occurred among the 190 patients who participated in the study.

### BTM changes

**Fig. 4:**
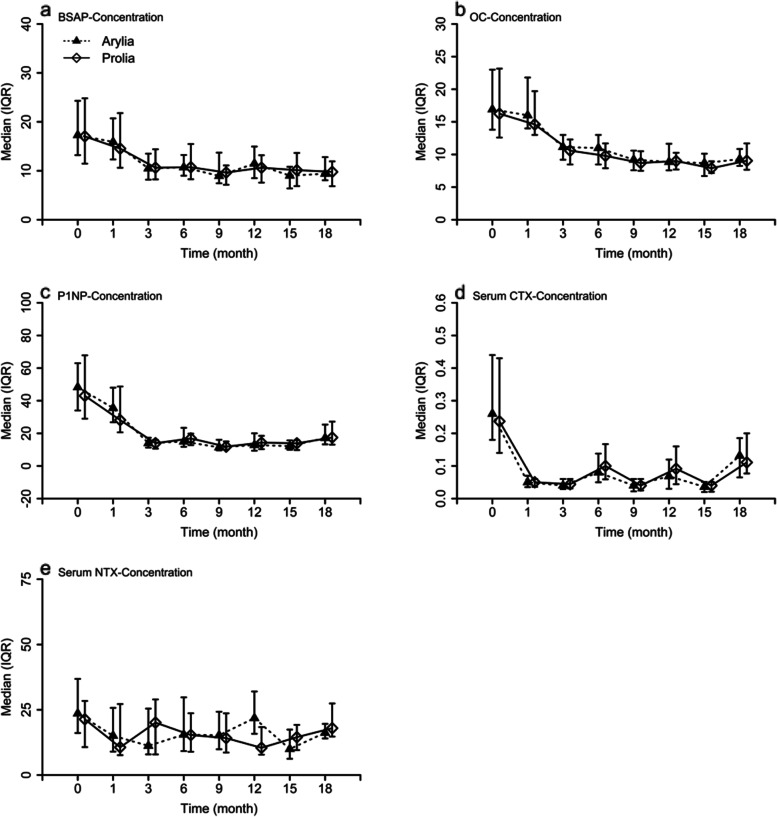
Medians and IQRs for biochemical markers of bone metabolism at 18 months of the trial

Changes in BTMs were not affected by the treatment group and followed a similar trend in both treatment groups. Bone formation markers, including BSAP, OC, and P1NP, and bone resorption markers, including CTX and NTX, all decreased over 18 months of the study (Fig. 4).

### Safety outcomes

In this study, a total of 135 AEs were reported. The most common AEs in both arms by system organ class (SOC) were metabolism and nutrition disorders and musculoskeletal and connective tissue disorders. Hypocalcemia and hypertension had the highest incidence among all AEs, and there were no significant differences between the Prolia® and Arylia arms.

The severity of AEs was evaluated using CTCAE v5.0. According to this classification, 119 AEs were categorized as grade 1 or 2, and 16 were categorized as grade 3. No grade 4 AEs were reported in this study. Among all AESIs, nine patients reported AEs in infections and infestations SOC, and three reported AEs in neoplasms benign, malignant, and unspecified (including cysts and polyps) SOC. Further details about these AEs are mentioned in Additional file 4. No report was found regarding eczema, ONJ, AFF, bone fracture, cardiovascular disorder, and pancreatitis acute.

Throughout this study, 13 serious adverse events (SAEs) were recorded in 12 patients, and all events resulted in patient hospitalization. All SAEs were considered unrelated to treatment. Additional file 4 demonstrates the summary of key safety results of the study.

### Immunogenicity

Among all the samples, two samples from months 0 and 6 were positive for one patient, and the samples from months 12 and 18 of this patient were negative. Other patient samples were negative for anti-denosumab antibodies at all time points.

## Discussion

In this study, the noninferiority of the biosimilar denosumab compared with the reference denosumab in improving BMD at the lumbar spine (L_1_–L_4_), total hip, and femoral neck among osteoporotic postmenopausal women was established. As in other studies [15, 16], the primary outcome can be determined by considering only the lumbar spine BMD since this site shows the highest incidence of osteoporosis, and the best results will be observed in short-term treatment compared to the total hip or femoral neck. Additionally, the primary outcome of a denosumab biosimilar candidate produced by Sandoz company is the assessment of BMD changes at the lumbar spine [17]. This ongoing phase 3 trial, which began after our study, shows that measuring BMD at the lumbar spine is sufficient for demonstrating bio-similarity.

In this study, in the PP set and after 18 months of treatment, the mean percentage change in BMD increased significantly in both treatment groups at the lumbar spine, total hip, and femoral neck. In the FREEDOM study [13], lumbar spine and total hip BMD increased in postmenopausal women with osteoporosis at 36 months of treatment with denosumab. In the study by Tomonori Kobayakawa and colleagues [18] comparing denosumab versus romosozumab in postmenopausal women with osteoporosis, the BMD at the lumbar spine, total hip, and femoral neck increased during 12 months of treatment in both groups. The increase in lumbar BMD was approximately two times that of the total hip and more than 2.5 times that of the femoral neck in the denosumab group. In another study, the percentage change in lumbar spine BMD with the biosimilar denosumab (Intas Pharmaceutical Ltd, India) was comparable to Prolia® and increased at 12 months of treatment in postmenopausal osteoporotic women [19]. In a study evaluating the efficacy of denosumab vs. teriparatide in glucocorticoid-induced osteoporotic patients, the BMD showed an increase at the lumbar spine and femoral neck; however, the percent change in the total hip BMD was not significant [20]. Overall, the results of our study on the percentage of BMD change in the three study sites are in line with the findings of the above studies, indicating an increase in BMD between 12 and 36 months of treatment.

Regarding the incidence of new vertebral fractures, both drugs were the same without any new vertebral fractures in patients. The role of denosumab in long-term fracture prevention is reviewed in an article published in 2020 [21]. Evaluation of fracture reduction with denosumab in postmenopausal osteoporosis in the FREEDOM study [13] showed that denosumab reduced the risk of new vertebral fractures, which is in line with the results of this study.

In addition, changes in biomarkers were not significantly different between the two groups, and the final values decreased relative to baseline. In a study conducted in postmenopausal patients in Argentina, changes in total alkaline phosphatase, OC, and serum CTX were downward during 12 months of treatment with denosumab regardless of previous bisphosphonate treatment [22]. In another study on postmenopausal women with osteoporosis in Japan [18], serum P1NP decreased at 6 and 12 months of treatment with denosumab. In the FREEDOM study [13], serum P1NP and CTX levels decreased during 36 months of treatment with denosumab. According to the above studies, a decreasing trend in BTMs has been observed since the early months of treatment with denosumab, indicating that such markers can be used for the early evaluation of effectiveness, and this trend has continued for up to 36 months, which is in accordance with the results of the present study.

Since osteoporosis is a chronic condition, safety concerns are a prerequisite for any treatment. Based on the trial findings, Arylia and Prolia® are generally comparable in terms of safety parameters. The overall incidence of AEs (77.89% and 64.21%, respectively, *p* = 0.26) and SAEs (6.32% and 6.32%, respectively, *p* = 1) were comparable between the two arms.

In this study, the most common adverse event was hypocalcemia (16.84% in the Arylia arm and 11.58% in the Prolia® arm) which was similar to another study that compared denosumab with zoledronic acid [23]. Additionally, the FREEDOM trial reported no difference in hypocalcemia incidence between the treated and placebo groups [24]. According to the FREEDOM study, denosumab can be associated with an increased risk of serious infections in women with postmenopausal osteoporosis. In the present study, reports by infections and infestations SOC were comparable between the Arylia and Prolia® arms (5.26% and 4.21%, respectively). In randomized, placebo-controlled trials comparing denosumab with placebo, more cases of neoplasm have been reported in the denosumab group compared with the placebo group by McClung et al. (1.9% versus 0%), Bone et al. (2.4% versus 0.6%), and in the FREEDOM trial (4.8% versus 4.2%) [25]. In this study, no cases of neoplasms occurred in the Arylia arm, compared to three cases in the Prolia® arm (*p* = 0.08). According to the investigators’ opinion, all the cases were unrelated to the treatment. There were no cases of ONJ and AFF in this study. There have been no reports of ONJ and AFF in osteoporosis clinical trials. Only in the fourth and fifth years of the FREEDOM extension trial were two cases of ONJ reported [24, 26].

A possible limitation of this study was the assessment duration. To evaluate rare and long-term AEs such as ONJ and new fractures, further studies with more prolonged periods and larger sample sizes are needed. In addition, a larger sample size can potentially detect more specific clinical differences between the two products.

## Conclusions

This study demonstrated the noninferiority of the biosimilar denosumab (Arylia) to the reference product in osteoporotic postmenopausal women. In general, there was no difference in the lumbar spine, total hip, or femoral neck BMD percentage change, the trend of bone metabolism biomarkers, or the occurrence of new vertebral fractures between the biosimilar denosumab and the reference product.

## Supplementary Information

**Additional file 1. **Comparison of the mean percentage change in BMD in the two treatment groups in the PP and ITT populations.

**Additional file 2.** Comparison of the mean percentage changes in BMD in the two treatment groups in the PP and ITT populations with the covariance analysis model.

**Additional file 3.** Forest plot for comparing Arylia versus Prolia® in terms of mean percent changes in BMD of the lumbar spine (L1-L4), total hip, and femoral neck in 18 months duration of the study. Forest plot demonstrating both t-test analysis and ANCOVA model for ITT set.

**Additional file 4.** Summary of the key safety results.

## Data Availability

The datasets used and/or analyzed during the current study are available from the corresponding author on reasonable request.

## Abbreviations

95% CI: 95% Confidence interval
AE: Adverse event
AESI: Adverse event of special interest
AFF: Atypical femoral fracture
BMD: Bone mineral density
BSAP: Bone-specific alkaline phosphatase
BTM: Bone turnover markers
CKD: Chronic kidney disease
CTCAE: Common Terminology Criteria for Adverse Events
CTX: C-terminal telopeptide
DXA: Dual-energy X-ray absorptiometry
FDA: Food and Drug Administration
FRAX: Fracture Risk Assessment Tool
GFR: Glomerular filtration rate
IOF: International Osteoporosis Foundation
ITT: Intention to treat
MedDRA: Medical Dictionary for Regulatory Activities
NTX: N-terminal telopeptide
OC: Osteocalcin
ONJ: Osteonecrosis of the jaw
P1NP: Procollagen type 1 N-terminal pro-peptide
PP: Per-protocol
RANK: Nuclear factor kappa-B
RANKL: Nuclear factor kappa-B ligand
SAE: Serious adverse events
SD: Standard deviation
SOC: System organ class
WHO: World Health Organization
