# Supplementary material for: Efficacy and safety of the biosimilar denosumab candidate (Arylia) compared to the reference product (Prolia®) in postmenopausal osteoporosis: a phase III, randomized, two-armed, double-blind, parallel, active-controlled, and noninferiority clinical trial
Source: Arthritis Res Ther. 2022 Jun 30;24:161. doi: 10.1186/s13075-022-02840-8 (PMC9245232; doi:10.1186/s13075-022-02840-8)
Supplement: Supplementary file 1 — Additional file 1. Comparison of the mean percentage change in BMD in the two treatment groups in the PP and ITT populations. [file 13075_2022_2840_MOESM1_ESM.docx]

| Population | Percentage of change in Arylia group | Percentage of change in Prolia^®^ group | Mean (confidence interval)^a^ | p-value* |
| --- | --- | --- | --- | --- |
|  | Mean±SD | Mean±SD |  |  |
| **PP population** | | | | |
| Spine BMD | 5.91±5.58 | 5.52±5.59 | 0.39 (-1.34,2.11) | 0.66 |
| Total hip BMD | 2.32±5.24 | 2.28±5.52 | 0.04 (-1.61,1.69) | 0.96 |
| Femoral Neck BMD | 1.91±6.32 | 1.50±6.62 | 0.41 (-1.58,2.40) | 0.68 |
| **ITT population** | | | | |
| Spine BMD | 5.80±5.43 | 5.54±5.40 | 0.26 (-1.34,1.87) | 0.75 |
| Total hip BMD | 2.05±5.74 | 2.29±5.37 | -0.24 (-1.89,1.40) | 0.77 |
| Femoral Neck BMD | 1.91±6.30 | 1.67±6.44 | 0.24 (-1.65,2.12) | 0.80 |

^a^Average percentage change of Arylia–Prolia^®^

*independent t-test
