# Supplementary material for: Efficacy and safety of the biosimilar denosumab candidate (Arylia) compared to the reference product (Prolia®) in postmenopausal osteoporosis: a phase III, randomized, two-armed, double-blind, parallel, active-controlled, and noninferiority clinical trial
Source: Arthritis Res Ther. 2022 Jun 30;24:161. doi: 10.1186/s13075-022-02840-8 (PMC9245232; doi:10.1186/s13075-022-02840-8)
Supplement: Supplementary file 2 — Additional file 2. Comparison of the mean percentage changes in BMD in the two treatment groups in the PP and ITT populations with the covariance analysis model. [file 13075_2022_2840_MOESM2_ESM.docx]

| Population | Arylia | | | Prolia^®^ | | | LS mean difference (95 % CI) | p-value* |
| --- | --- | --- | --- | --- | --- | --- | --- | --- |
|  | **LS mean** | **SE** | **95% CI** | **LS mean** | **SE** | **95% CI** |  |  |
| **PP population** | | | | | | | | |
| Spine BMD | 5.89 | 0.60 | (4.70,7.09) | 5.53 | 0.62 | (4.31,6.75) | 0.36 (-1.35,2.07) | 0.67 |
| Total hip BMD | 2.28 | 0.54 | (1.21,3.36) | 2.31 | 0.55 | (1.21,3.40) | -0.02 (-1.56,1.51) | 0.97 |
| Femoral Neck BMD | 1.94 | 0.67 | (0.62,3.27) | 1.46 | 0.68 | (0.12,2.81) | 0.48 (-1.41,2.37) | 0.62 |
| **ITT population** | | | | | | | | |
| Spine BMD | 5.82 | 0.56 | (4.71,6.93) | 5.52 | 0.58 | (4.38,6.66) | 0.30 (-1.29,1.89) | 0.71 |
| Total hip BMD | 2.06 | 0.53 | (1.01,3.12) | 2.27 | 0.55 | (1.19,3.35) | -0.21 (-1.71,1.30) | 0.79 |
| Femoral Neck BMD | 1.99 | 0.62 | (0.76,3.22) | 1.59 | 0.64 | (0.33,2.84) | 0.40 (-1.36,2.16) | 0.65 |

* Based on ANCOVA model with the presence of therapeutic group and adjusted BMD baseline.
