# Supplementary figures and images for: Efficacy and safety of the biosimilar denosumab candidate (Arylia) compared to the reference product (Prolia®) in postmenopausal osteoporosis: a phase III, randomized, two-armed, double-blind, parallel, active-controlled, and noninferiority clinical trial
Source: Arthritis Res Ther. 2022 Jun 30;24:161. doi: 10.1186/s13075-022-02840-8 (PMC9245232; doi:10.1186/s13075-022-02840-8)

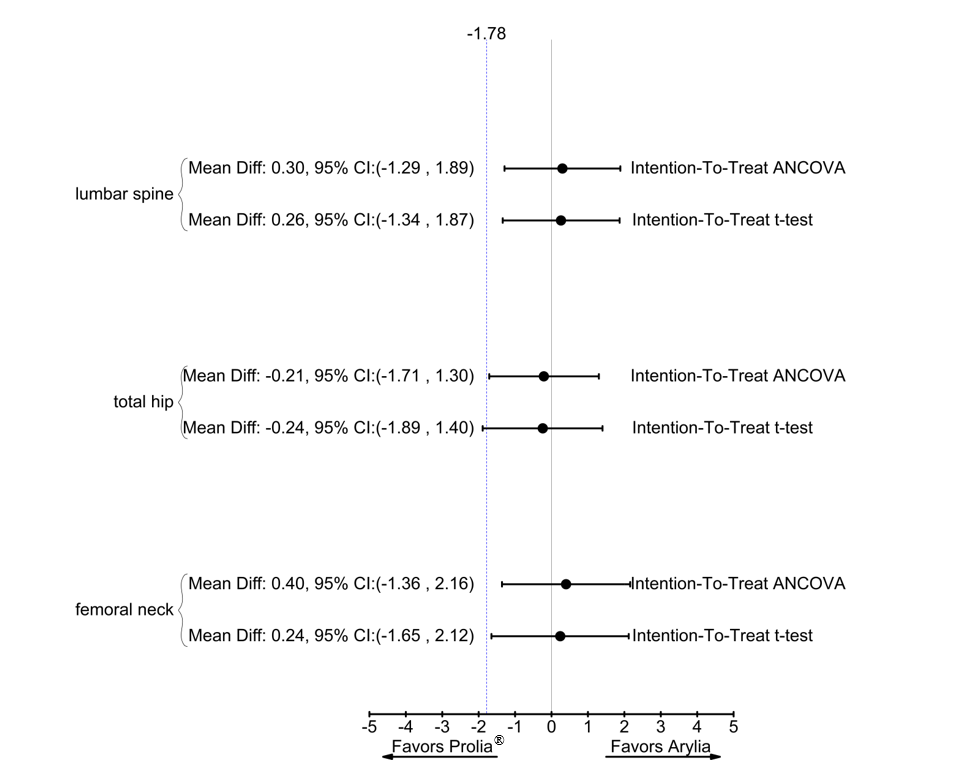

Supplement: Supplementary file 3 — Additional file 3. Forest plot for comparing Arylia versus Prolia® in terms of mean percent changes in BMD of the lumbar spine (L1-L4), total hip, and femoral neck in 18 months duration of the study. Forest plot demonstrating both t-test analysis and ANCOVA model for ITT set. [file 13075_2022_2840_MOESM3_ESM.docx]
