# Supplementary material for: Efficacy and safety of the biosimilar denosumab candidate (Arylia) compared to the reference product (Prolia®) in postmenopausal osteoporosis: a phase III, randomized, two-armed, double-blind, parallel, active-controlled, and noninferiority clinical trial
Source: Arthritis Res Ther. 2022 Jun 30;24:161. doi: 10.1186/s13075-022-02840-8 (PMC9245232; doi:10.1186/s13075-022-02840-8)
Supplement: Supplementary file 4 — Additional file 4. Summary of the key safety results. [file 13075_2022_2840_MOESM4_ESM.docx]

|  | **Arylia (n=95)** | **Prolia^®^(n=95)** | **p-value*** |
| --- | --- | --- | --- |
| All adverse events | 74 (77.89) | 61 (64.21) | 0.26 |
| Serious adverse events | 6 (6.32) | 6 (6.32) | 1 |
| At least possibly related adverse events | 22 (23.16) | 24 (25.26) |  |
| **AESI (SOCs)** ^a^ | | | |
| Infections and infestations | 5 (5.26) | 4 (4.21) | 0.73 |
| Neoplasms benign, malignant and unspecified (incl cysts and polyps) | 0 (0) | 3 (3.16) | 0.08 |
| **Adverse events occurring in at least 2% of subjects** | | | |
| Arthralgia | 3 (3.16) | 1 (1.05) |  |
| Back pain | 2 (2.11) | 4 (4.21) |  |
| Hypertension | 7 (7.37) | 3 (3.16) |  |
| Hypertriglyceridaemia | 3 (3.16) | 2 (2.11) |  |
| Hypocalcaemia | 16 (16.84) | 11 (11.58) |  |
| Hypophosphataemia | 0 (0) | 3 (3.16) |  |
| Muscle spasms | 1 (1.05) | 2 (2.11) |  |
| Musculoskeletal pain | 1 (1.05) | 2 (2.11) |  |
| Nasopharyngitis | 2 (2.11) | 1 (1.05) |  |
| Osteoarthritis | 2 (2.11) | 0 (0) |  |
| Pyuria | 2 (2.11) | 0 (0) |  |
| Rash | 3 (3.16) | 1 (1.05) |  |
| Data are n (%). | | | |
| *Based on Chi-squared test.  ^a^Adverse events of special interest (system organ classes) | | | |
